# Co-assembly of dipeptide and hydrophobic drug in hyaluronic acid through Schiff base reaction for the treatment of osteoarthritis

**DOI:** 10.1016/j.pscia.2026.100133

**Published:** 2026-07-04

**Authors:** Guixin Chen, Qin Li, Chen Yu, Hao Liu, Xueping Guo, Aoli Wu, Xiaoming Zhang, Hengchang Zang

**Affiliations:** a NMPA Center for Innovation and Research in Regulatory Science, School of Pharmaceutical Sciences, Cheeloo College of Medicine, Shandong University, Jinan, Shandong, 250012, China; b Department of Pharmacy, Qilu Hospital of Shandong University, Jinan, Shandong, 250012, China; c Bloomage GAG Biotechnology (Shenzhen) Corporation Limited, Shenzhen, Guangdong, 518107, China; d School of Science, Optoelectronics Research Center, Minzu University of China, Beijing, 100081, China; e State Key Laboratory of Discovery and Utilization of Functional Components in Traditional Chinese Medicine, Jinan, Shandong, 250012, China

**Keywords:** Hyaluronic acid, Cationic dipeptide, Schiff base, Co-assembly, Osteoarthritis, Hydrophobic drugs

## Abstract

The development of multiple drugs for the synergistic osteoarthritis (OA) therapy is essential, particularly in light of the limited efficacy of existing clinical interventions. However, the poor bioavailability and systemic toxicity of hydrophobic drugs and their incompatibility with hydrophilic components largely hindered the co-delivery of multi-drugs. In this study, we present a dynamic covalent assembly strategy within a hyaluronic acid (HA) matrix to fabricate cationic dipeptide-based carrier particle (CGCP/HA). This carrier is designed to encapsulate hydrophobic nonsteroidal anti-inflammatory drug (NSAID) celecoxib and IL-1β inhibitor diacerein via a one-step co-assembly method, facilitating targeted drug delivery and combination therapy for OA. The resulting particles demonstrated exceptional stability, injectability, and biocompatibility in vitro, while mitigating the adverse effects associated with conventional oral OA medications. In a rat OA model, intra-articular administration of these co-assembled materials significantly suppressed inflammatory cytokines (TNF-α, IL-1β, and PGE2) and demonstrated enhanced cartilage repair by integrating multiple functions of hydrophilic HA, supramolecular carriers, and hydrophobic drugs. This work established a simple strategy for co-assembly of biomolecules and hydrophobic drug molecules and holds significant clinical promise for the treatment of OA.

## Highlights

• Dynamic covalent assembly enables co-delivery of hydrophobic OA drugs.
• Combination of dipeptide and HA improve stability and injectability of carrier.
• Co-assembled particles reduce systemic toxicity of celecoxib and diacerein.
• Intra-articular delivery suppresses inflammation and enhances cartilage repair.

## Graphical abstract

**Figure:**
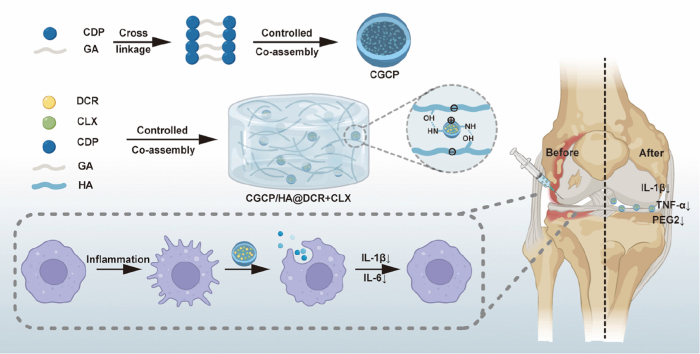

**Table:** List of Abbreviations

Abbreviations	Definition
CCK-8	Cell counting kit-8
CDP	Cationic diphenylalanine
CLSM	Confocal Laser Scanning Microscope
CLX	Celecoxib
DLS	Dynamic light scattering
DCR	Diacerein
DMEM	Dulbecco's modified eagle medium
DMSO	Dimethyl sulfoxide
D_2_O	Deuterium oxide
EDS	Energy Spectroscopy
EE	Entrapment efficiency
ELISA	Enzyme linked immunosorbent assay
FBS	Fetal bovine serum
FT-IR	Fourier transform infrared
GA	Glutaraldehyde
HA	Hyaluronic acid
H&E	Hematoxylin and Eosin staining
^1^H NMR	hydrogen nuclear magnetic resonance spectroscopy
HPLC	High performance liquid chromatography
IL-6	Interleukin-6
LE	Loading efficiency
LPS	Lipopolysaccharide
NSAIDs	non-steroidal anti-inflammatory drugs
OA	Osteoarthritis
ROS	Reactive oxygen species
SEM	Scanning electron microscope
TB	Toluidine Blue
TEM	Transmission electron microscope
TNF-α	Tumor necrosis factor-α
UV-Vis	Ultraviolet-visible
XPS	X-ray photoelectron spectroscopy

## 1 Introduction

Osteoarthritis (OA) is a prevalent degenerative joint disease characterized by joint pain, limited mobility, and cartilage degeneration, which poses a significant global health burden [[1], [2], [3], [4], [5]]. Current treatment modalities, including oral non-steroidal anti-inflammatory drugs (NSAIDs) [[6], [7], [8]], often provide limited symptomatic relief and are hampered by poor bioavailability, potential systemic side effects (e.g., gastrointestinal discomfort), and inability to reverse established articular cartilage lesions.

Intra-articular therapeutic injection offers a localized alternative, which allows precise drug delivery to the affected joint and minimizing systemic exposure [9,10]. Hyaluronic acid (HA), a key component of synovial fluid recognized for its excellent compatibility with biological tissues, has been extensively utilized in the intra-articular injections to alleviate OA symptoms [[11], [12], [13]]. HA exerts functions in targeting chondrocyte CD44 receptors, joint lubrication, pain alleviation, and mobility improvement [[14], [15], [16]]. However, its efficacy is typically transient, and limitations in stability, integration of other functional drugs, and sustained-release properties within the joint microenvironment impede long-term therapeutic performance. However, merely relying on hyaluronic acid is insufficient to meet the treatment requirements for osteoarthritis [17,18]. Consequently, there is an urgent need to explore innovative delivery systems that can achieve efficient drug encapsulation, localized sustained release, and reduced side effects, thereby improving treatment outcomes and enhancing patient prognosis.

Combining HA with other anti-inflammatory agents is critical, as it can synergistically address inflammation, lubrication deficiency, and cartilage repair [[19], [20], [21], [22]]. This combinatorial approach will outperform single-drug formulations in preclinical models, with enhanced therapeutic efficacy. However, they struggle to meet the core need for low-dose combination therapy synergy and the clinical requirement for multi-drug combination therapy in OA due to the distinct physicochemical properties of multi-drug therapeutic materials [[23], [24], [25], [26]]. Supramolecular assemblies based on dynamic covalent chemistry have advanced significantly in drug delivery systems [[27], [28], [29]]. Schiff bases, due to their mild reaction conditions and pollution-free by-product, have the capacity to form biofriendly carrier materials through the reaction of short peptides and small crosslinking agent [[30], [31], [32]]. These carriers can effectively protect the active ingredient, thereby enhancing its stability within biological systems and enabling controlled release. This innovative drug delivery system not only improves the bioavailability of pharmaceuticals but also facilitates the simultaneous release of multiple therapeutic agents [[33], [34], [35], [36]]. Therefore, combining the advantages of supramolecular drug delivery carriers with those of hyaluronic acid is expected to synergistic treatment for OA.

**Scheme 1:**
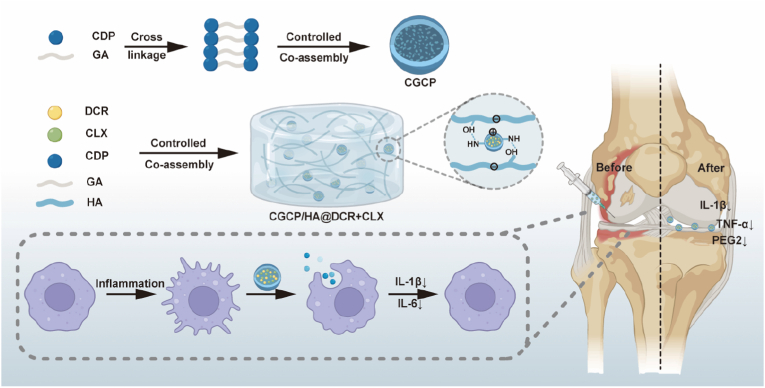
Schematic illustration of the preparation and therapeutic application of CGCP/HA@DCR + CLX for OA.

In this study, we developed an injectable drug delivery system for OA treatment by dynamic covalent assembly of cationic diphenylalanine (CDP) within HA (Scheme 1). This system co-encapsulates two hydrophobic drugs, celecoxib (CLX, a COX-2 inhibitor) and diacerein (DCR, an IL-1β inhibitor), to synergize anti-inflammatory, chondroprotective, and analgesic effects while providing lubrication and visco-supplementation. The resulting particles exhibited excellent colloidal stability, injectability, and biocompatibility, which effectively enhancing drug solubility and reducing adverse effects of conventional oral OA drugs. In a rat OA model, intra-articular administration of CGCP/HA@DCR + CLX suppressed inflammatory cytokines (TNF-α, IL-1β, PGE2) and promoted cartilage repair. This approach holds substantial clinical promise for OA treatment and could effectively overcome key limitations of conventional therapies.

## 2 Materials and methods

### 2.1 Materials

HA (MW = 780 kDa) was obtained from Bloomage Biotechnology Co., Ltd. CDP was purchased from GL Biochem (Shanghai) Ltd. Glutaraldehyde (GA, 25% in H_2_O) was purchased from Sigma-Aldrich Trading Co., Ltd. CLX and DCR were purchased from Shanghai Aladdin Biochemical Technology Co., Ltd. Nile red (NR, 97.5%) was purchased from J&K Scientific Co., Ltd. Thioflavin T (ThT, 98%) was purchased from Innochem (Beijing) Technology Co., Ltd. The mouse embryonic fibroblasts (NIH-3T3) cells and the mouse mononuclear macrophages (RAW 264.7) cells were provided from Shanghai Fuheng Biotechnology Co., Ltd. Dulbecco's modified Eagle medium (DMEM) and fetal bovine serum (FBS) were purchased from Invitrogen/Gibco Biotechnology Co., Ltd. Lipopolysaccharide (LPS) and L-Cysteine were purchased from Beijing Solarbio Science & Technology Co., Ltd. All reagents were used directly without further purification. Ultrapure water (18.2 MΩ cm) was used throughout the experiments.

### 2.2 Sample preparation

#### 2.2.1 Preparation of CDP-GA carrier particles (CGCP)

1 mg of CDP was precisely weighed and dissolved in 500 μL H_2_O. 3.6 μL of GA solution, with a mass fraction of 12.5%, was added to the mixture, resulting in the formation of the precipitate after one day of aging at room temperature. To remove any residual GA, the precipitate was subjected to three cycles of centrifugation with ultrapure water. It was then stored in ultrapure water at room temperature for subsequent experiments.

#### 2.2.2 Preparation of CGCP/HA

1 mg of CDP was dissolved in 500 μL H_2_O. Subsequently, HA was weighed according to the molar ratios of CDP to HA, specifically 1:0.5, 1:1, 1:2, 1:4, and 1:6. The HA was then incorporated into the CDP solution. Once the HA was completely dissolved, 3.6 μL of GA solution, with a mass fraction of 12.5%, was added to the mixture. The mixture was aged for one day at room temperature. To eliminate excess GA, the crude CGCP/HA underwent dialysis in ultrapure water for a duration of one day, utilizing a dialysis bag with a molecular weight cut-off (MWCO) of 3500 Da. During this dialysis process, the water was replaced four times. The final solution was then freeze-dried to obtain the lyophilized powder of CGCP/HA, which was subsequently stored in a refrigerator at 4 °C.

### 2.3 Characterizations

#### 2.3.1 ^1^H NMR

The ^1^H NMR spectra of the samples after dissolved in D_2_O were recorded using a Bruker AVD 400 MHz spectrometer.

#### 2.3.2 Fourier transform infrared (FTIR) spectroscopy

The FTIR spectroscopy of samples after freeze drying were characterized by a Bruker ALPHA II infrared spectrometer at room temperature.

#### 2.3.3 Ultraviolet-Visible (UV-Vis) absorption spectroscopy

The UV-Vis absorption spectra of the samples were recorded at room temperature using a Cary 60 UV-Vis spectrophotometer with a quartz cuvette of 1 mm optical range.

#### 2.3.4 Transmission electron microscopy (TEM) imaging

A pipette of the samples was transferred onto a copper grid and excess liquid was carefully removed from the edges of the copper grid using absorbent paper. Once the drying process was complete, the sample was analyzed using a JEOL JEM-1230 transmission electron microscope.

#### 2.3.5 Scanning electron microscope (SEM) imaging

A pipette of samples were deposited onto a clean silicon wafer. After dried and coated with platinum, the samples were examined under a SEM (JSM-7610FPlus) to assess its dimensions and morphology.

#### 2.3.6 Dynamic light scattering (DLS)

Zeta potential analyses were conducted using a Malvern Nano ZS instrument, with distilled water employed as the dispersing medium. 1 mL of the sample was pipetted into the zeta potential cell, and the temperature was maintained at 25.0 °C.

#### 2.3.7 Fluorescence (FL) spectroscopy

The FL spectra of the samples were recorded using a Cary Eclipse fluorescence spectrophotometer with a quartz cuvette of 1 mm optical range.

#### 2.3.8 Confocal laser scanning microscope (CLSM)

Multi-channel images of the samples were collected using a Leica TCS SP8 STED ultra-high resolution confocal microscope with excitation light excitation at 405 nm (emission range 430-490 nm), 488 nm (emission range 500-570 nm), and 561 nm (emission range 590-690 nm), respectively.

#### 2.3.9 Energy spectroscopy (EDS)

The SEM samples were imaged using a TESCAN MIRA LMS SEM, operating at an accelerating voltage of 3 kV for topographical analysis and 15 kV for energy spectrum acquisition, with a SE2 secondary electron detector.

### 2.4 Drug-carrying properties of CGCP/HA

The DCR/CLX loading ratio was set at 0.8:1 according to the clinically established combination therapy used in OA treatment [[37], [38], [39], [40]]. Similar to the preparation of CGCP/HA, the DCR and CLX were incorporated into CGCP/HA using the co-precipitation method. Initially, HA and CDP were dissolved in distilled water. Subsequently, 100 μL of DMSO solution containing DCR (0.8 mg mL^−1^) and CLX (1 mg mL^−1^) was added. After mixing, 3.6 μL of the GA solution was incorporated. The CGCP/HA@DCR, CGCP/HA@CLX, and CGCP/HA@DCR + CLX were obtained after a one-day aging at room temperature. To eliminate any residual GA, DMSO, and unincorporated drugs, the samples underwent a 24-h dialysis process utilizing a dialysis bag with a molecular weight cut-off (MWCO) of 3500 Da. Subsequently, the samples were freeze-dried and stored in a refrigerator at 4 °C for future use.

The drug loading was quantified using high-performance liquid chromatography (HPLC, Agilent 1220 Infinity II). The chromatographic column utilized was an Agilent ZORBAX SB-C18 (250 mm × 4.6 mm, 5 μm). The mobile phase consisted of an aqueous solution of glacial acetic acid (designated as phase A) and methanol (designated as phase B), with the pH adjusted to 2.0. The flow rate was maintained at 1.0 mL/min, and the ultraviolet detector was calibrated to a wavelength of 254 nm at a temperature of 25.0 °C, with an injection volume of 20 μL. The separation of components was achieved through a gradient elution method, the details of which are outlined below. The following formula is used for the calculation of drug loading (DL) and encapsulation rate (EE):

DL% = (*W*_*T*_ -*W*_*F*_ / *W*_*NP*_) × 100%

EE% = (*W*_*T*_ -*W*_*F*_ / *W*_*T*_) ×100%

where W_T_ is the amount of drug administered, W_F_ is the amount of free drug, and W_NP_ is the total mass of drug-carrying particles.

### 2.5 Drug release behavior of CGCP/HA@DCR + CLX

Initially, phosphate buffer saline solutions (PBS) with pH values of 5.5 and 7.4 were prepared. The CGCP/HA@DCR + CLX prepared in the above experiments were collected and centrifuged at 8000 rpm for 5 min. After discarding the supernatant, fixed volumes of PBS buffer solution with different pH values were added to the precipitate. The specific preparation of the medium for CXL and DCR release was PBS solution containing 0.5% Tween 80. The resulting mixture was incubated in a constant temperature shaker at 37 °C at a shaking speed of 100 rpm. At predetermined time intervals, 1 mL of supernatant was taken out for HPLC analysis, and immediately replenished with equal volume of fresh PBS buffer solution to maintain the total volume of the system. Finally, according to the standard curve of drug concentration and absorbance, HPLC absorption spectroscopy was used to quantify the drug release in the supernatant at each time point. Parallel experiments were conducted for each group (*n* = 3).

### 2.6 Biological activity evaluations

#### 2.6.1 In vitro cell viability assay

The biocompatibility of the samples was evaluated using the CCK-8 assay. Briefly, NIH-3T3 cells were seeded into 96-well plates at a density of 8000 cells per well and incubated at 37 °C in a 5% CO_2_ atmosphere for 24 h. After incubation, the culture medium was removed, and the cells were gently washed once with sterile PBS. The blank control group received high-glucose complete DMEM, while the other groups were treated with DMEM containing different concentrations of the samples (100 μL per well) and further incubated for 24 h. Following this, the medium was discarded, and 100 μL of 10% CCK-8 solution was added to each well, followed by thorough mixing. The absorbance of each well was measured at 450 nm using a microplate reader. Cell viability was calculated as follows:

Cell viability (%) = (OD_s_ – OD_b_) / (OD_c_ − OD_b_) × 100%

OD_s_: Experimental wells (containing cells, samples to be tested, CCK-8). OD_b_: Blank wells (without cells and samples to be tested, CCK-8). OD_c_: Control wells (with cells, CCK-8, without samples to be tested).

#### 2.6.2 Establishment of cellular inflammation model

RAW 264.7 cells were cultured in high-glucose DMEM complete medium. Upon reaching the logarithmic growth phase, the effects of various concentrations of CGCP/HA (0, 50, 100, 200, and 300 μg mL^−1^) on cell viability were first assessed. Subsequently, a cellular inflammation model was established by lipopolysaccharide (LPS) induction. Briefly, RAW 264.7 cells were seeded into 12-well plates at a density of 3 × 10^5^ cells per well and incubated in a temperature-controlled incubator for 24 h. The original medium was then discarded, and basal medium was added for treatment and induction over an additional 24 h period. The cells were divided into the following groups: negative control (NC) group, LPS-induced group, low-dose group (LPS + 50 μg mL^−1^ CGCP/HA@DCR + CLX), and high-dose group (LPS + 500 μg mL^−1^ CGCP/HA@DCR). The NC group was maintained in serum-free medium, while all other groups were maintained in basal medium containing 1 μg mL^−1^ LPS.

#### 2.6.3 Detection of TNF-α in cell supernatants

Following a 24-h treatment period, the cell supernatant was collected and centrifuged at 1000 × g for 20 min to remove impurities. Subsequently, the supernatant was transferred to a 1.5 mL centrifuge tube. The concentration of TNF-α in the RAW 264.7 supernatant was measured using the methodology outlined in the enzyme-linked immunosorbent assay (ELISA) kit provided by Jiangsu Jingmei Biotechnology Co., Ltd.

### 2.7 Anti-OA research

#### 2.7.1 Establishment of rat OA models

The rat model of OA studied was established utilizing a non-surgical pain phenotype modeling method. The animal study protocol was approved by the Animal Care and Use Committee (ACUC) of the Ethical Committee of the School of Pharmaceutical Sciences, Shandong University (No. YXDW2025-0036). Wistar rats (SPF grade) were randomly assigned to six groups, which are designated as follows: a normal control group (Normal), a model group (Model), a blank preparation group [CGCP/HA), a free drug group (DCR + CLX (Free)], a low HA-containing drug-carrying preparation group (CGCP/1HA@DCR + CLX), and a high HA-containing drug-carrying preparation group (CGCP/4HA@DCR + CLX). Each group comprised three rats, with each specimen assigned a unique numerical identifier. Following a one-week acclimatization period, the body weight and right knee joint width of the rats were recorded. The rats were anesthetized via intraperitoneal injection of 3% sodium pentobarbital (40 mg kg^−1^) and subsequently underwent right knee deconditioning. Except for the Normal group, 50 μL of a solution containing 10% papain and L-cysteine (0.03 mol L^−1^) was administered into the joint cavity of the right knee on days 1, 4, and 7. Before each modeling session, the body weights and knee widths of the rats were recorded, and the OA model was established two weeks following the initial injection.

#### 2.7.2 Articular injection therapy with drug-containing carrier particles

Once the OA model was established, the treatment regimen consisted of weekly injections of the relevant medication over a duration of two weeks. The specific protocols were delineated as follows: the Normal group did not receive any treatment; the Model group was administered 50 μL of PBS into the joint cavity; the CGCP/HA group received 50 μL of CGCP/HA injected into the joint cavity. The DCR + CLX (Free) group was treated with 50 μL of DCR + CLX (including 16 mg mL^−1^ DCR and 20 mg mL^−1^ CLX) into the joint cavity, while the CGCP/1HA@DCR + CLX group received 50 μL of CGCP/1HA@DCR + CLX via joint cavity injection. Additionally, the CGCP/4HA@DCR + CLX group was administered 50 μL of CGCP/4HA@DCR + CLX through joint cavity injection. Before each administration, the width of the right knee joint and the body weight of the rats were measured.

#### 2.7.3 Detection of inflammatory cytokines in rat serum

One week after drug administration, blood samples were collected from rats via the inner canthus vein. The serum was subsequently isolated through centrifugation at 2000 rpm for 25 min at a temperature of 4 °C, following 30 min of natural coagulation at room temperature. The concentrations of TNF-α, IL-1β, and PGE2 in the rat serum were analyzed using ELISA. Since the primary objective of this assay was to examine differences among the various groups and identify significant differences, no standard curve was established, and the results were normalized.

#### 2.7.4 Micro-CT scanning of the rat knee

The rats were euthanized through cervical dislocation, after which the right knee joint was excised. Surrounding muscle and other tissues were meticulously removed to the greatest extent possible, and the intact knee joint was subsequently placed in 4% paraformaldehyde for fixation. Micro-CT scans of the rat knee joints were performed using a Quantum GX2 small animal in vivo tomography system. The scanning parameters included a voltage of 90 kV, a current of 88 μA, a dose of 927 mGy, a rotation angle of 270°, and a high-resolution scanning mode with a duration of 4 min. Following the acquisition of the original images, the knee joints were promptly returned to 4% paraformaldehyde, and the data were then transferred to the Micro-CT data workstation for three-dimensional reconstruction of the knee joints.

#### 2.7.5 Histopathological examinations

To evaluate the therapeutic effects of various treatments on OA, knee tissues were re-fixed following Micro-CT scanning, and gradient decalcification was conducted using an EDTA solution. Subsequently, the tissues underwent a series of histological analyses, which included hematoxylin and eosin (H&E) staining, Safranin O-Fast Green staining, and immunohistochemical analyses for TNF-α, COX-2, and COL2. Following these procedures, the knee tissues were processed for paraffin embedding and sectioning.

Semi-quantitative histological scoring of H&E− and Safranin O/Fast Green-stained sections was performed by three blinded observers using a modified Mankin scoring system, with higher scores indicating more severe cartilage degeneration and proteoglycan loss. The mean score of each sample was used for statistical analysis. Immunohistochemical staining was quantified using Image-Pro Plus 6.0 by measuring the integrated optical density (IOD) and positive staining area of each section, and the average optical density was calculated as:

AOD = IOD / area

#### 2.7.6 Safety evaluation of drug-containing carrier particles

To evaluate the safety of drug-carrying formulations in rats, organs known to potentially exhibit toxicity, including the heart, liver, spleen, lungs, and kidneys, were fixed in 4% paraformaldehyde. These organs were then embedded in paraffin and sectioned at a thickness of 4 μm per slice. The resulting sections were stained with H&E and subsequently visualized and photographed using a VS120 panoramic digital sectioning and scanning microscope.

#### 2.7.7 Statistical analysis

The data were expressed as mean ± standard deviation (SD). The data were subjected to one-way analysis of variance (ANOVA) using the statistical software package SPSS. The symbols ∗, ∗∗, and ∗∗∗ indicate statistical significance at the 0.05, 0.01, and 0.001 levels, respectively. A *p*-value of 0.05 or less was considered statistically significant.

## 3 Results and discussion

### 3.1 Preparation and characterization of CGCP and CGCP/HA

CDP-GA carrier particle (CGCP) was prepared through a Schiff base reaction between CDP and GA, resulting in the formation of compounds that subsequently underwent self-assembly. The mechanism of the Schiff base reaction was illustrated in Fig. S1. Simultaneously, the CGCP/HA was produced through the Schiff base reaction between CDP and GA within HA solution, utilizing the non-covalent interactions between HA and CDP. To evaluate the impact of HA on the assembly of CDP-GA, different concentrations of HA were added to prepare CGCP/nHA. Fig. S2 presented macroscopic images of CGCP and CGCP/nHA. After this period, the CGCP solution displayed a light-yellow coloration, accompanied by the formation of precipitates. In contrast, the CGCP/HA solution exhibited a gradual transition from light yellow to milky white as the ratio of HA increased. This transition was characterized by a significant reduction in particle aggregation, resulting in a homogeneous solution devoid of any noticeable precipitation. The addition of HA was found to enhance the dispersion of carrier particles and colloidal solution with stability and injectability were obtained.

**Fig. 1:**
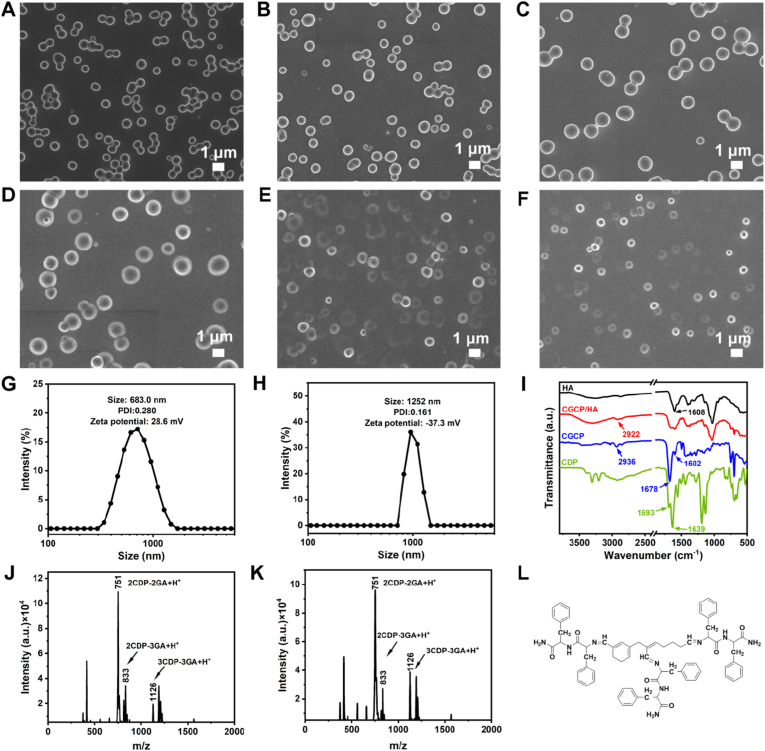
The SEM images of (A) CGCP, (B) CGCP/0.5HA, (C) CGCP/1HA, (D) CGCP/2HA, (E) CGCP/4HA, and (F) CGCP/6HA. Size distribution and zeta potential of (G) CGCP and (H) CGCP/1HA measured by DLS. (I) FTIR spectra of HA, CGCP/HA, CDP-GA, and CDP. The MALDI-TOF-MS spectra of (J) CGCP and (K) CGCP/HA. (L) The chemical structure of 3CDP-3GA.

To comprehensively investigate the influence of HA content on the morphology and size of the CGCP/HA assemblies, complementary electron microscopy techniques were employed. SEM imaging (Fig. 1A–F) and subsequent statistical analysis (Fig. S3) revealed that the particle size of the spherical assemblies could be effectively regulated by varying the HA content. Specifically, the average particle size increased from 815 nm to 1425 nm as the molar ratio of CDP to HA changed from 1:0.5 to 1:2, but decreased upon a further increase to a 1:6 ratio. TEM analysis (Fig. S4) corroborated the spherical morphology with smooth surfaces for both CGCP and CGCP/HA, confirming that the core structure of CGCP was preserved through non-covalent interactions (hydrogen bonds) even in the presence of HA. At elevated HA concentrations, increased solution viscosity led to some blurring in TEM images, yet the overall morphology remained discernible.

This non-monotonic size variation is attributed to the synergistic effect of electrostatic interactions and solution viscosity. DLS and zeta potential measurements (Fig. 1G and H) provided further mechanistic insights. The incorporation of HA dramatically reversed the zeta potential of CGCP from +28.6 mV to −37.3 mV for CGCP/1HA, indicating that the positively charged amino groups on CGCP electrostatically interacted with the carboxylic acid groups of HA, forming negatively charged complexes. The higher absolute zeta potential value of CGCP/HA also contributes to its enhanced colloidal stability and reduced aggregation propensity. At lower HA concentrations, enhanced electrostatic cross-linking between CGCP and HA leads to particle growth. Conversely, at higher HA concentrations, the electrostatic repulsion from excess HA polymers, coupled with increased solution viscosity that hinders assembly, collectively results in a reduction of particle size. All systems were classified as single dispersions, underscoring the effectiveness of this preparation method.

FTIR spectroscopy was employed to elucidate the molecular-level assembly mechanism of CGCP and CGCP/HA by characterizing the specific chemical bonds involved. As shown in Fig. 1I, the FTIR spectra of CDP, CGCP, HA, and CGCP/HA were systematically compared. The CDP spectrum exhibited a characteristic amide I band in the range of 1630-1700 cm^−1^, attributable primarily to C=O stretching vibrations, suggesting that CDP predominantly adopts a parallel β-sheet secondary structure. In contrast, the spectrum of CGCP displayed a marked enhancement of the absorption peak at 1678 cm^−1^ accompanied by a pronounced decrease at 1602 cm^−1^. These spectral changes indicate a structural reorganization into a predominant antiparallel β-sheet conformation within the CGCP assembly framework [41], underscoring the role of hydrogen bonding in stabilizing the supramolecular architecture.

The assembly mechanism is governed by two key interactions: covalent Schiff base formation and non-covalent co-assembly with HA. First, the covalent framework of CGCP is confirmed by the relative enhancement of the 2936 cm^−1^ band, assigned to the C–H stretch of the Schiff base formed between CDP and GA [41], despite the overlapping C=N signal. Second, the integration of HA is verified by a distinct red-shift of the 2922 cm^−1^ peak in the CGCP/HA composite, compared to the characteristic HA carboxylate peak at 1608 cm^−1^. This shift signifies a non-covalent co-assembly process, most likely through intermolecular hydrogen bonding that weakens the bond force constant and lowers the vibrational frequency. These findings collectively substantiate that the final architecture is stabilized by both covalent cross-links and HA-mediated supramolecular interactions.

The molecular configuration of the assembly unit of CGCP and CGCP/HA were investigated using MALDI-TOF-MS. As illustrated in Fig. 1J and K, three distinct peaks at *m*/*z* values of 751, 833, and 1126 were identified as 2CDP-2GA+H^+^, 2CDP-3GA+H^+^, and 3CDP-3GA+H^+^, respectively. The chemical structures of the three assembly motifs were illustrated in Figure S1 and Fig. 1L. This finding indicated that CDP binds to the aldehyde group of GA oligomers formed through a hydroxyl-aldehyde condensation reaction. The CGCP were formed through the assembly of three CDP-GA motifs. Furthermore, the presence of HA does not appear to influence the Schiff base reaction of CDP with GA, as evidenced by the persistence of molecular ion peaks at *m*/*z* 751, 833, and 1126 in the CGCP/HA system.

**Fig. 2:**
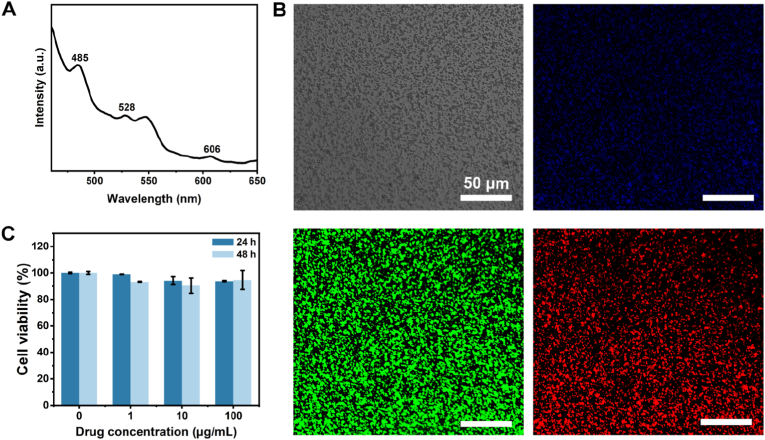
(A) The fluorescence emission spectra of CGCP/HA were obtained by laser excitation at a wavelength of 405 nm. (B) Bright-field CLMS plots for CGCP/HA, and blue (430-490 nm), green (500-570 nm) and red (590-690 nm) CLSM plots were collected in multiple channels under excitation at 405 nm, 488 nm and 561 nm, respectively. Scale bars, 50 μm. (C) The in vitro cell viability of NIH-3T3 was determined by a CCK-8 assay in an environment containing CGCP/HA (*n* = 3). (For interpretation of the references to color in this figure legend, the reader is referred to the Web version of this article.)

Supramolecular assemblies can exhibit synergistic properties distinct from their individual components. Since the formation of C=N bonds via Schiff base reaction can confer intrinsic autofluorescence, we investigated the fluorescence characteristics of the CGCP/HA assemblies. Upon excitation at 405 nm, the CGCP/HA particles exhibited emission peaks at 485, 528, and 606 nm (Fig. 2A). This multi-color fluorescence profile was corroborated by CLSM, which detected distinct signals in the blue (430-490 nm), green (500-570 nm), and red (590-690 nm) channels when excited at 405, 488, and 561 nm, respectively (Fig. 2B). This autofluorescence originates from the n-π∗ transition of the Schiff base bonds (C=N) [42]. The label-free nature of this fluorescence circumvents potential interference from external dyes, positioning CGCP/HA as a promising tracer material for biological applications.

In order to investigate the biocompatibility of the assemblies, CGCP/HA particles (at concentrations of 0, 1, 10, and 100 μg mL^−1^, respectively) were co-cultured with NIH/3T3 cells for 24 and 48 h, after which time they were evaluated by the CCK-8 method. As illustrated in Fig. 2C, the NIH/3T3 cells exhibited robust growth following treatment with varying concentrations of CGCP/HA particles. The cell activity remained above 90% for 24 and 48 h, suggesting that the CGCP/HA particles possess favorable biocompatibility and may serve as a promising drug carrier.

### 3.2 CGCP/HA drug loaded and in vitro anti-inflammation

**Fig. 3:**
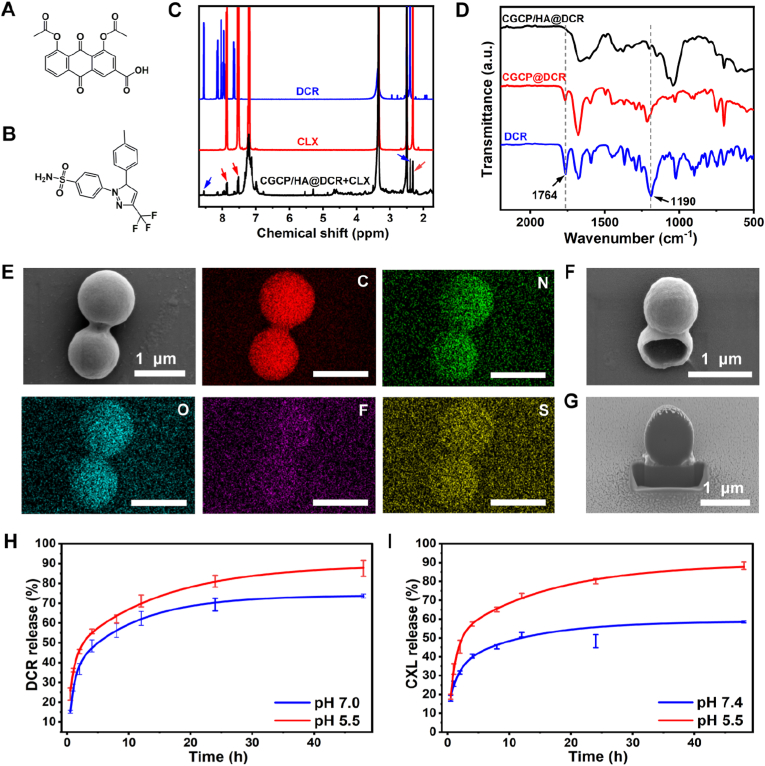
The structural formulas of (A) DCR and (B) CLX. (C) The ^1^H NMR spectra of DCR, CLX, and CGCP/HA@DCR + CLX. (D) The FTIR spectra for CGCP@DCR, CGCP/HA@DCR, and DCR. (E) The EDS test for CGCP/HA@DCR + CLX. The elemental mappings for carbon C, nitrogen N, oxygen O, fluorine F, and S. (F) SEM image of the fragmented CGCP/HA, obtained through ultrasonic crushing. (G) SEM image of CGCP/HA@DCR + CLX cut by ion beam. Scale bars, 1 μm. The release curves of (H) DCR and (I) CLX in CGCP/HA@DCR + CLX at different pH values.

The integration of multifunctional molecules into supramolecular assemblies holds considerable potential for expanding their utility in areas such as targeted therapy, probe development, and controlled drug delivery. To evaluate the drug-loading capacity of CGCP/HA carrier particles, we selected model dyes and drugs with distinct solubility profiles: water-soluble ThT and hydrophobic NR, as well as the therapeutic agents DCR and CLX. Visual evidence of successful encapsulation was provided by the distinct colored precipitates observed after centrifugation (Fig. S5). Specifically, DCR and CLX were loaded in a controlled manner, yielding green and white precipitates, respectively, indicating the formation of CGCP/HA@DCR and CGCP/HA@CLX complexes. The successful encapsulation of both Diacerein (DCR, Fig. 3A) and celecoxib (CLX, Fig. 3B) within the CGCP/HA assembly was unequivocally confirmed by ^1^H NMR spectroscopy (Fig. 3C). The spectrum of the co-loaded system (CGCP/HA@DCR + CLX) exhibited characteristic proton signals assignable to each drug: for DCR, the bis-acetyl and bis-phenyl protons at *δ* 2.40 and 8.56 ppm, respectively; and for CLX, the methyl and phenyl protons at *δ* 2.32, 7.53, and 7.87 ppm. These distinct resonances not only verify the effective incorporation of both drug molecules but also underscore the capability of the CGCP/HA carrier to accommodate pharmacologically diverse agents through a controlled loading process.

To elucidate the drug encapsulation mechanism within the CGCP/HA carrier, we first employed FTIR spectroscopy using DCR as a model drug (Fig. 3D). The characteristic peaks of DCR (C–O stretch at 1190 cm^−1^ and C=O stretch at 1764 cm^−1^) remained evident in the spectrum of CGCP@DCR, indicating that a portion of the drug was located on the particle surface. In contrast, these distinctive DCR peaks were absent in the spectrum of CGCP/HA@DCR, suggesting that the drug was entirely encapsulated within the CGCP/HA matrix without surface exposure.

Subsequently, SEM imaging revealed that the co-loaded CGCP/HA@DCR + CLX particles retained a uniform spherical morphology with smooth surfaces (Fig. S6), confirming that drug encapsulation did not compromise the structural integrity of the assemblies. Furthermore, EDS mapping confirmed the homogeneous distribution of F and S elements, intrinsic to CLX, throughout the particles (Fig. 3E). This provides direct evidence for the successful and uniform encapsulation of the model drugs.

To elucidate the structural basis for the high drug-loading capacity, the internal morphology of the particles was examined. Carrier particles with an internal cavity typically offer a larger specific surface area and higher volumetric drug capacity compared to dense or surface-adsorbent carriers. To probe the internal morphology, CGCP/HA particles were subjected to ultrasonication, which revealed a substantial hollow structure upon rupture (Fig. 3F). This observation was further validated using a FIB to cross-section the particles. FIB analysis clearly showed that pristine CGCP/HA possesses a defined hollow cavity (Fig. S7), which became predominantly filled in the drug-loaded CGCP/HA@DCR + CLX particles (Fig. 3G). This morphological transition directly demonstrates the remarkable drug accommodation capacity of the internal cavity. In summary, CGCP/HA particles exhibit a stable, uniform spherical morphology with an internal hollow structure that confers a robust ability to encapsulate guest molecules. This combination of attributes makes these assemblies highly promising for biomedical applications, particularly in drug delivery.

The drug loading capacity of the CGCP/HA carrier was quantitatively evaluated using HPLC, following established methodologies [43,44]. The analysis was performed at a detection wavelength of 254 nm for DCR, CLX, and their mixture. Under the optimized chromatographic conditions, both drugs were well-resolved with excellent peak shapes, a separation factor greater than 1.5, and a theoretical plate number exceeding 5000. DCR and CLX exhibited distinct retention times at approximately 10 and 20 min, respectively, which remained consistent across measurements (Fig. S8). The CGCP/HA particles demonstrated high encapsulation efficiency and drug loading for both agents: 88.30% ± 1.12% and 3.25% ± 0.16% for DCR, 96.35% ± 2.36% and 4.06% ± 0.32% for CLX, respectively. The total encapsulation efficiency and drug loading reached 94.23% ± 1.75% and 6.89% ± 0.23%, indicating that the CGCP/HA system can efficiently co-load two poorly soluble drugs with a promising loading capacity [45].

The drug release profiles of CGCP/HA@DCR + CLX were evaluated under physiological conditions (pH 7.4 and pH 5.5). As shown in Fig. 3H and I, both DCR and CLX exhibited significantly accelerated release in acidic environments compared with neutral conditions, reaching a plateau after approximately 24 h. After 48 h, the cumulative release of DCR was 87.54 ± 4.01% at pH 5.5 and 73.64 ± 0.94% at pH 7.4. For CLX, the corresponding release values were 88.34 ± 2.04% and 58.48 ± 0.49% at pH 5.5 and pH 7.4, respectively. This pH-responsive release behavior is particularly advantageous for OA therapy, as it enables enhanced and site-specific drug delivery within the acidic inflammatory microenvironment.

**Fig. 4:**
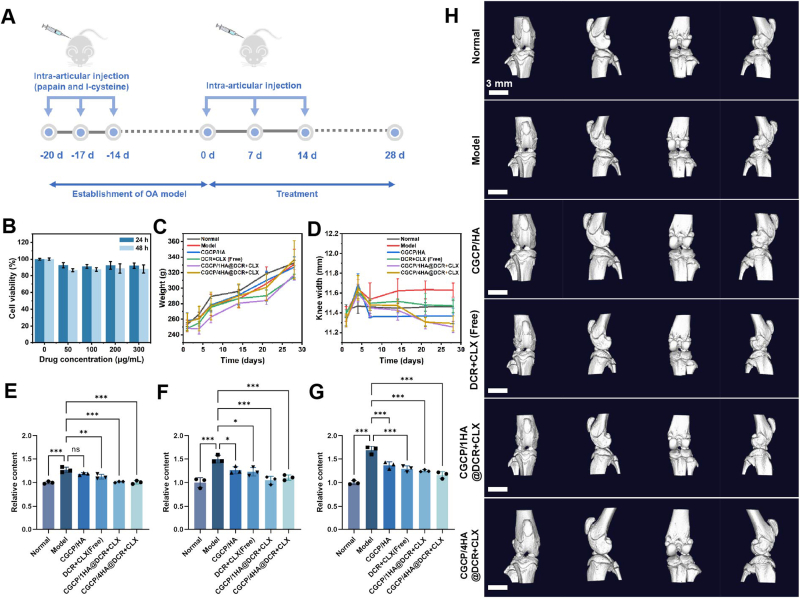
Therapeutic effects of CGCP/HA@DCR + CLX in OA rat models. (A) Schematic illustration of the experimental treatment in Wistar rats with OA. (B) RAW264.7 cell viability after exposure to different concentrations of CGCP/HA (n = 3). (C) Body weight and (D) knee width changes of rats in different groups (n = 3). Groups: Normal, Model, CGCP/HA, DCR + CLX (Free), CGCP/1HA@DCR + CLX, and CGCP/4HA@DCR + CLX. (E) Serum TNF-α, (F) IL-1β, and (G) PGE2 levels in different groups (n = 3). (H) Representative micro-CT 3D images of rat knee joints. Scale bar, 3 mm.

To investigate the effect of drug-loaded particles on the LPS-induced inflammation model using RAW 264.7 cells, it was essential to assess the cytotoxicity of these particles on RAW 264.7. This preliminary step was necessary to ensure that subsequent experimental results would not be influenced by cell viability (Fig. 4A). As shown in Fig. 4B, the RAW 264.7 cells demonstrated robust growth and maintained a survival rate exceeding 85% after treatment with varying concentrations of CGCP/HA particles for 24 and 48 h. This finding indicates that the CGCP/HA are essentially non-toxic to the cells and will not compromise the results of subsequent experiments.

The levels of the cytokine TNF-α in the supernatant of RAW 264.7 cells treated with drug-loaded particles were assessed using an ELISA. As shown in Fig. S9, the levels of TNF-α in the supernatant of RAW 264.7 cells stimulated by LPS were significantly elevated. In contrast, treatment with varying concentrations of CGCP/HA@DCR + CLX resulted in a reduction of TNF-α levels. These values were significantly different from those observed in the LPS-only induced group, suggesting that CGCP/HA@DCR + CLX can inhibit the secretion of TNF-α by RAW 264.7 cells and may have the potential to alleviate inflammation.

### 3.3 Evaluation of the in vivo therapeutic effect of CGCP/HA@DCR + CLX

To evaluate the systemic and local responses to OA induction and treatment, rat body weight and knee joint width were measured throughout the study (Fig. 4C and D). Following model establishment, all OA groups exhibited lower body weight compared to the normal group, indicating an adverse systemic effect on health. After treatment, rats administered with drug-loaded formulations showed a significant recovery in body weight. Concurrently, a notable increase in knee joint width was observed in all OA groups post-modeling, consistent with inflammatory swelling. Treatment with drug-loaded carriers resulted in a more pronounced reduction in joint width compared to other groups, suggesting effective alleviation of local inflammation.

To further evaluate the systemic anti-inflammatory effects of the treatments, serum levels of key pro-inflammatory mediators (TNF-α, IL-1β, and PGE2) were quantified via ELISA (Fig. 4E–G). All treatment groups exhibited reduced cytokine levels compared to the model group. Notably, the reductions were significantly more pronounced in the groups receiving drug-loaded particles (CGCP/1HA@DCR + CLX and CGCP/4HA@DCR + CLX) than in those treated with the blank carrier (CGCP/HA) or free drugs (DCR + CLX), demonstrating the superior efficacy of the formulated systems in suppressing systemic inflammation.

Furthermore, micro-CT analysis of rat knee joints provided structural insights into the therapeutic outcomes (Fig. 4H). The normal group displayed a smooth articular surface without pathological bone formation. In stark contrast, the model group exhibited marked structural deterioration, characterized by a roughened joint surface, increased osteophyte formation, and the presence of free bone tissue, thereby validating the successful induction of OA. Treatment with blank CGCP/HA or free drugs partially ameliorated these changes, slightly reducing surface roughness and osteophyte formation. However, the most significant structural recovery was observed in the groups treated with the drug-loaded carriers (CGCP/HA@DCR + CLX), which showed markedly smoother articular surfaces and an absence of osteoid and free bone tissue, closely resembling the normal joint architecture. This indicates that the co-delivery system possesses robust efficacy in promoting cartilage repair and restoring joint integrity.

**Fig. 5:**
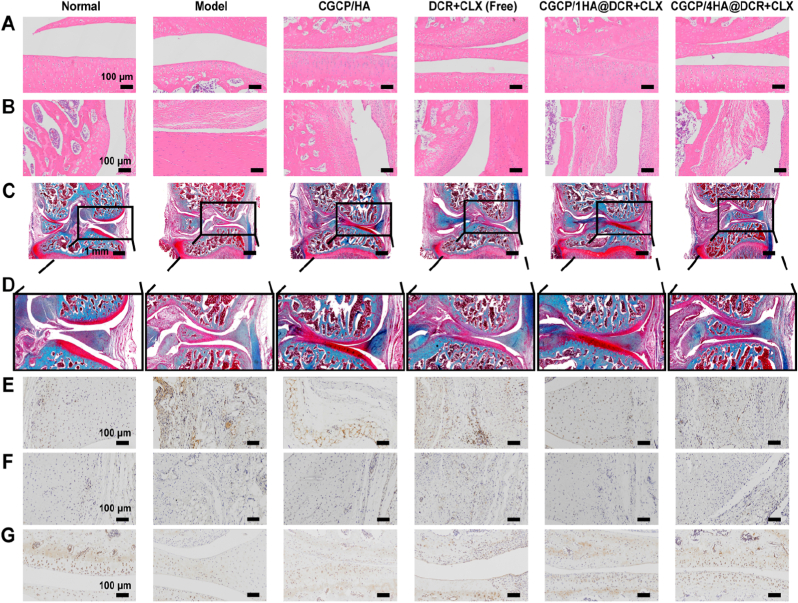
Representative images of (A) the cartilage area and (B) the synovial area of H&E stained sections of rat knee joints. Scale bars, 100 μm. (C) Representative images of Safranin O-fast green stained sections of knee joints from rats. Scale bars, 1 mm. (D) Local magnification of (C). Images of immunohistochemical staining for (E) TNF-α, (F) COX-2, and (G) COL2 in the synovial region of rat knee joints. Groups (*n* = 3): Normal, Model, CGCP/HA, DCR + CLX (Free), CGCP/1HA@DCR + CLX, and CGCP/4HA@DCR + CLX. Scale bars, 100 μm. (For interpretation of the references to color in this figure legend, the reader is referred to the Web version of this article.)

To assess cartilage integrity and synovial inflammation, knee joint sections were subjected to H&E staining. As shown in Fig. 5A, the cartilage structure in the control group exhibited a smooth surface, a thick cartilage layer, and a high density of well-organized chondrocytes. In stark contrast, the model group displayed severe cartilage degradation, characterized by a rough and irregular surface, marked thinning of the cartilage layer, and a reduced number of disorganized chondrocytes.

Histological analysis of H&E-stained sections provided further evidence of the therapeutic efficacy (Fig. 5A and B). The scoring results were shown in Fig. S10A. In the cartilage region, treatment with blank CGCP/HA carrier or free drugs resulted in only modest improvements, including a slightly smoother surface and a modest increase in cartilage thickness relative to the model group. In contrast, the joints treated with the drug-loaded formulation (CGCP/4HA@DCR + CLX) exhibited more substantial cartilage restoration. In the synovial region, the normal group displayed a loose, cell-sparse structure without inflammatory infiltration. The model group, however, showed severe synovitis, characterized by massive inflammatory cell infiltration and pronounced synovial cell hyperplasia, consistent with the observed joint swelling. Notably, the groups receiving the drug-loaded preparations demonstrated a remarkable resolution of synovitis, with no discernible inflammatory infiltration, reduced synovial hyperplasia, and an absence of vascular proliferation. In conclusion, the intra-articular injection of drug-loaded carriers elicited a superior anti-inflammatory and tissue-reparative outcome compared to the free drug.

Safranin O-fast green staining was performed to evaluate the proteoglycan content and structural integrity of cartilage following OA induction and treatment (Fig. 5C and D). The scoring results were shown in Fig. S10B. In the control group, the cartilage structure remained intact with intense and extensive Safranin O staining, reflecting abundant proteoglycan deposition. In contrast, the model group exhibited substantial cartilage erosion in the femoral region, loss of Safranin O staining, lighter fast green staining, and signs of osteoporosis, confirming successful OA modeling.

Treatment with blank CGCP/HA carrier or free drugs (DCR + CLX) partially restored cartilage integrity in the tibial region, but failed to reverse proteoglycan loss or osteoporotic changes in the femur. Notably, the therapeutic outcomes were significantly enhanced in the drug-loaded groups (CGCP/1HA@DCR + CLX and CGCP/4HA@DCR + CLX), which displayed well-preserved cartilage architecture, intense Safranin O staining, and markedly improved subchondral bone structure. The sustained drug release from the carrier system, combined with the intrinsic bioactivity of HA, likely contributed to this comprehensive reparative effect. In conclusion, the CGCP/HA-based co-assembly system not only served as an efficient drug carrier but also exerted a synergistic role in OA therapy. The HA component contributed to lubricating the joint and mitigating inflammation, while the sustained co-delivery of DCR and CLX from the hollow carrier structure enabled long-lasting anti-inflammatory and cartilage-protective effects. These results strongly support that the CGCP/HA@DCR + CLX formulation represents a promising combined strategy for the treatment of osteoarthritis.

Despite the widespread clinical use of oral NSAIDs for OA management, their therapeutic efficacy is substantially limited by systemic adverse effects, particularly cardiotoxicity [46]. To evaluate the potential systemic toxicity of the developed CGCP/HA@DCR + CLX formulation, histopathological examinations were performed on major organs (heart, liver, spleen, lungs, and kidneys) in an OA rat model.

**Fig. 6:**
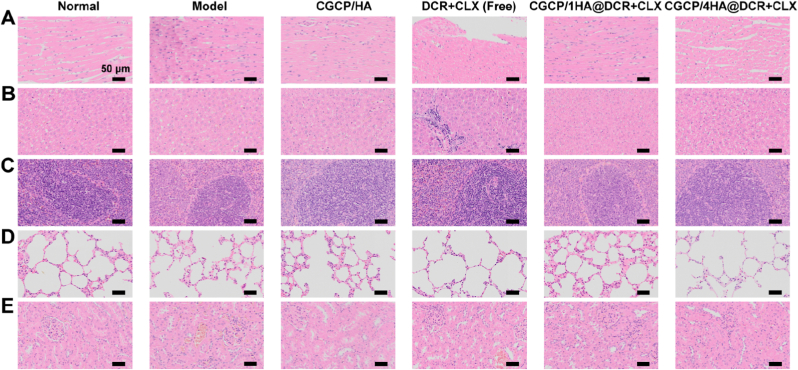
H&E staining of (A) heart section, (B) liver section, (C) spleen section, (D) lung section, and (E) kidney section. Scale bars, 50 μm.

As illustrated in Fig. 6A–H&E staining of cardiac tissues from the free DCR + CLX group revealed fibrin deposition in cardiac chambers and collagen fiber proliferation in subendocardial and interstitial regions. In contrast, the CGCP/HA@DCR + CLX-treated groups maintained normal cardiac architecture with well-defined endocardial, myocardial, and epicardial structures, and exhibited uniformly stained, well-aligned cardiac myofibers. Analysis of other organs (Fig. 6B–E) showed mild lymphocyte infiltration in the hepatic periportal area only in the free drug group, while no significant pathological alterations were observed in other treatment groups. These findings demonstrate that the CGCP/HA-based co-delivery system effectively mitigates the systemic toxicity associated with free drugs while maintaining an excellent safety profile, highlighting its potential as a promising long-acting therapeutic strategy for OA.

Notably, this study has some limitations and needs further improvement. Although the co-assembly strategy improved drug solubility and therapeutic performance, the precise in vivo fate, degradation behavior, and long-term metabolism of the supramolecular carriers require further investigation. Secondly, the synergistic mechanisms between HA, CDP, and the co-loaded drugs need to be further elucidated at the molecular level. In addition, the current evaluation was mainly based on in vitro studies and a rat OA model. Further validation in large animal models and clinical studies is required to confirm its long-term safety, efficacy, and translational feasibility.

## 4 Conclusions

In conclusion, this study illustrates that the CGCP/HA co-assembly system provides a versatile and biocompatible strategy for OA therapy. Its design enables efficient co-delivery of multiple hydrophobic drugs while maintaining structural stability, auto-fluorescently, and injectability. The CGCP/HA@DCR + CLX formulation demonstrated pronounced anti-inflammatory efficacy and cartilage reparative potential in vivo, without inducing significant organ toxicity. These findings underscore the potential of such supramolecular carriers to advance minimally invasive OA treatment and highlight its promise as a safe and effective strategy for clinical OA management.

## Data availability

The Data will be made available on request.

## Ethics approval

The animal study protocol was approved by the Animal Care and Use Committee (ACUC) of the Ethical Committee of the School of Pharmaceutical Sciences, Shandong University (No. YXDW2025-0036).

## Declaration of generative AI in scientific writing

No generative AI tools have been used throughout the entire writing process of this manuscript.

## Funding information

This research was supported by financial support from the 10.13039/501100001809National Natural Science Foundation of China (22572108, 12574240), National Key R&D Program of China (2024YFC3506900, 2024YFC3506902); the Future Scholar Program of Shandong University, and the Young Talent of Lifting engineering for Science and Technology in Shandong, China (SDAST2025QTA059).

## CRediT authorship contribution statement

**Guixin Chen:** Conceptualization, Investigation, Methodology, Writing – original draft. **Qin Li:** Investigation, Writing – original draft, Writing – review & editing. **Chen Yu:** Investigation, Methodology. **Hao Liu:** Investigation, Writing – review & editing. **Xueping Guo:** Methodology. **Aoli Wu:** Conceptualization, Funding acquisition, Methodology, Supervision, Writing – review & editing. **Xiaoming Zhang:** Writing – review & editing. **Hengchang Zang:** Funding acquisition, Supervision.

## Declaration of competing interest

The authors declare that they have no known competing financial interests or personal relationships that could have appeared to influence the work reported in this paper.

## References

[1] Coryell P.R., Diekman B.O., Loeser R.F. (2021). Mechanisms and therapeutic implications of cellular senescence in osteoarthritis. Nat. Rev. Rheumatol..

[2] Martel-Pelletier J., Barr A.J., Cicuttini F.M., Conaghan P.G., Cooper C., Goldring M.B., Goldring S.R., Jones G., Teichtahl A.J., Pelletier J.P. (2016). Osteoarthritis. Nat. Rev. Dis. Primers.

[3] Zhang Y., Jiao X., Wang T., Yue X., Wang Y., Cai B., Wang C., Lu S. (2024). piRNA mmu_piR_037459 suppression alleviated the degeneration of chondrocyte and cartilage. Int. Immunopharmacol..

[4] Zhang D., Zhang Y., Xia S., Shen P., Yang C. (2024). Metabolic profiling of synovial fluid in human temporomandibular joint osteoarthritis. Front. Immunol..

[5] Zhang Y., Zhang D., Liao X., Xu Q., Bu L., Zheng J., Shen P., Yang C. (2025). Novel insights into the role of ferroptosis in temporomandibular joint osteoarthritis and knee osteoarthritis. Int. J. Med. Sci..

[6] Duong V., Oo W.M., Ding C., Culvenor A.G., Hunter D.J. (2023). Evaluation and treatment of knee pain: a review. JAMA.

[7] Cho Y., Jeong S., Kim H., Kang D., Lee J., Kang S.B., Kim J.H. (2021). Disease-modifying therapeutic strategies in osteoarthritis: current status and future directions. Exp. Mol. Med..

[8] Zhang Y., Zhang D., Xu Q., Xia S., Shen P., Yang C. (2025). Fostamatinib alleviates temporomandibular joint osteoarthritis by maintaining cartilage homeostasis through MAPK/NF-κB and AKT/mTOR pathways. Int. Immunopharmacol..

[9] Li G., Liu S., Chen Y., Zhao J., Xu H., Weng J., Yu F., Xiong A., Udduttula A., Wang D., Liu P., Chen Y., Zeng H. (2023). An injectable liposome-anchored teriparatide incorporated gallic acid-grafted gelatin hydrogel for osteoarthritis treatment. Nat. Commun..

[10] Xu X., Liang Y., Li X., Ouyang K., Wang M., Cao T., Li W., Liu J., Xiong J., Li B., Xia J., Wang D., Duan L. (2021). Exosome-mediated delivery of kartogenin for chondrogenesis of synovial fluid-derived mesenchymal stem cells and cartilage regeneration. Biomaterials.

[11] George E. (1998). Intra-articular hyaluronan treatment for osteoarthritis. Ann. Rheum. Dis..

[12] Walvekar P., Lulinski P., Kumar P., Aminabhavi T.M., Choonara Y.E. (2024). A review of hyaluronic acid-based therapeutics for the treatment and management of arthritis. Int. J. Biol. Macromol..

[13] Marinho A., Nunes C., Reis S. (2021). Hyaluronic acid: a key ingredient in the therapy of inflammation. Biomolecules.

[14] Chang W., Chen L., Chen K. (2024). The bioengineering application of hyaluronic acid in tissue regeneration and repair. Int. J. Biol. Macromol..

[15] Luo Z., Dai Y., Gao H. (2019). Development and application of hyaluronic acid in tumor targeting drug delivery. Acta Pharm. Sin. B.

[16] Yin Y., Zhou H., Liu K., Wu A., Zang H. (2025). Hyaluronic acid-dissolving microneedles integrated with multienzyme-like nanozymes for treating drug-resistant bacteria-infected wounds. Int. J. Biol. Macromol..

[17] Nabizadeh Z., Nasrollahzadeh M., Heidari F., Nasrabadi D. (2024). A drug-loaded nano chitosan/hyaluronic acid hydrogel system as a cartilage tissue engineering scaffold for drug delivery. Int. J. Biol. Macromol..

[18] Li H., Guo H., Lei C., Liu L., Xu L., Feng Y., Ke J., Fang W., Song H., Xu C., Yu C., Long X. (2019). Nanotherapy in joints: increasing endogenous hyaluronan production by delivering hyaluronan synthase 2. Adv. Mater..

[19] Yang Y., Zhao X., Wang S., Zhang Y., Yang A., Cheng Y., Chen X. (2023). Ultra-durable cell-free bioactive hydrogel with fast shape memory and on-Demand drug release for cartilage regeneration. Nat. Commun..

[20] Yao Q., Chen X., Sheng H., Zhang Y., Chen R., Fan P., Kou L. (2025). Lubricating nano/micro particles for osteoarthritis therapy. Mater. Horiz..

[21] Luan Z., Ma X., Zhao Q., Yang A., Li J. (2024). “Integrated Diagnosis and Treatment” of upconversion nanocomposite hydrogel for osteoarthritis treatment. Chem. Eng. J..

[22] Mao X., Yang Y., Qian Y., Chen K., Zhao Y., Shang S., Zhang H. (2025). Hydration‐lubricated, cartilage‐targeted, and anti‐inflammatory triple‐functional nanospheres for the treatment of osteoarthritis. Adv. Funct. Mater..

[23] Yang Y., Zhang H. (2024). Intra-articular injection of nanomaterials for the treatment of osteoarthritis: from lubrication function restoration to cell and gene therapy. Adv. Funct. Mater..

[24] Chen J., Du C., Tang B., Liu J., Xiao P., Wang X., Li Z.A., Huang W., Lei Y. (2025). Application and progress of smart hydrogel microspheres for regulating oxidative stress in osteoarthritis. Chem. Eng. J..

[25] Lin J., Jia S., Cao F., Huang J., Chen J., Wang J., Liu P., Zeng H., Zhang X., Cui W. (2024). Research progress on injectable microspheres as new strategies for the treatment of osteoarthritis through promotion of cartilage repair. Adv. Funct. Mater..

[26] Sang X., Zhao X., Yan L., Jin X., Wang X., Wang J., Yin Z., Zhang Y., Meng Z. (2022). Thermosensitive hydrogel loaded with primary chondrocyte-derived exosomes promotes cartilage repair by regulating macrophage polarization in osteoarthritis tissue. Eng. Regen. Med..

[27] Ding Y., Hu X., Piao Y., Huang R., Xie L., Yan X., Sun H., Li Y., Shi L., Liu Y. (2023). Lipid prodrug nanoassemblies via dynamic covalent boronates. ACS Nano.

[28] Li Q., Wu A., Zhang M., Zhang X., Zang H. (2024). Adaptive covalently assembled thymopentin/hyaluronic acid based anti-inflammatory drug carrier with injectability and controlled release. Int. J. Biol. Macromol..

[29] Webber M.J., Langer R. (2017). Drug delivery by supramolecular design. Chem. Soc. Rev..

[30] Wu A., Guo Y., Li X., Li Q., Chen G., Zang H., Li J. (2023). Schiff base nanoarchitectonics for supramolecular assembly of dipeptide as drug carriers. J. Colloid Interface Sci..

[31] Jia Y., Li J. (2015). Molecular assembly of schiff base interactions: construction and application. Chem. Rev..

[32] Sun B., Tao K., Jia Y., Yan X., Zou Q., Gazit E., Li J. (2019). Photoactive properties of supramolecular assembled short peptides. Chem. Soc. Rev..

[33] Chang Y., Jiao Y., Symons H.E., Xu J.F., Faul C.F., Zhang X. (2019). Molecular engineering of polymeric supra-amphiphiles. Chem. Soc. Rev..

[34] Isaacs L. (2012). Approaches to drug delivery based on the principles of supramolecular chemistry. Adv. Drug Deliv. Rev..

[35] Wang J.T.W., Rodrigo A.C., Patterson A.K., Hawkins K., Aly M.M.S., Sun J., Al Jamal K.T., Smith D.K. (2021). Enhanced delivery of neuroactive drugs via nasal delivery with a self-healing supramolecular gel. Adv. Sci..

[36] Qin Y., Tong F., Zhang W., Zhou Y., He S., Xie R., Lu L. (2021). Self‐delivered supramolecular nanomedicine with transformable shape for ferrocene‐amplified photodynamic therapy of breast cancer and bone metastases. Adv. Funct. Mater..

[37] Singh S., Jain A., Mishra S.K., Singh S., Singh R. (2012). Estimation of chondroitin sulfate as a biomarker in articular cartilage for treatment of osteoarthritis through oral diacerein loaded lipid nanoparticles in rat model. Osteoarthr. Cartil..

[38] Khandelia R., Hodgkinson T., Crean D., Brougham D.F., Scholz D., Ibrahim H., Quinn S.J., Rodriguez B.J., Kennedy O.D., O'Byrne J.M., Brayden D.J. (2024). Reproducible synthesis of biocompatible albumin nanoparticles designed for intra-articular administration of celecoxib to treat osteoarthritis. ACS Appl. Mater. Interfaces.

[39] Xiang H., Zhang C., Xiong Y., Wang Y., Pu C., He J., Chen L., Jiang K., Zhao W., Yang H., Wang F., Li Y. (2024). MMP13-Responsive hydrogel microspheres for osteoarthritis treatment by precise delivery of celecoxib. Mater. Des..

[40] Jung J.H., Kim S.E., Kim H.J., Park K., Song G.G., Choi S.J. (2020). A comparative pilot study of oral diacerein and locally treated diacerein-loaded nanoparticles in a model of osteoarthritis. Int. J. Pharm..

[41] Zhang H., Fei J., Yan X., Wang A., Li J. (2015). Enzyme-responsive release of doxorubicin from monodisperse dipeptide-based nanocarriers for highly efficient cancer treatment in vitro. Adv. Funct. Mater..

[42] Khan S., Chen X., Almahri A., Allehyani E.S., Alhumaydhi F.A., Ibrahim M.M., Ali S. (2021). Recent developments in fluorescent and colorimetric chemosensors based on schiff bases for metallic cations detection: a review. J. Environ. Chem. Eng..

[43] Giannellini V., Salvatore F., Bartolucci G., Coran S.A., Bambagiotti-Alberti M. (2005). A validated HPLC stability-indicating method for the determination of diacerhein in bulk drug substance. J. Pharm. Biomed. Anal..

[44] El-Kafrawy D.S., Abo-Gharam A.H., Abdel-Khalek M.M., Belal T.S. (2022). Comparative study of two versatile multi-analyte chromatographic methods for determination of diacerein together with four non-steroidal anti-inflammatory drugs: greenness appraisal using analytical eco-scale and AGREE metrics. Sustain. Chem. Pharm..

[45] Shen S., Wu Y., Liu Y., Wu D. (2017). High drug-loading nanomedicines: progress, current status, and prospects. Int. J. Nanomed..

[46] Dubreuil M., Louie-Gao Q., Peloquin C.E., Choi H.K., Zhang Y., Neogi T. (2018). Risk of myocardial infarction with use of selected non-steroidal anti-inflammatory drugs in patients with spondyloarthritis and osteoarthritis. Ann. Rheum. Dis..

