# Supplementary material for: Co-assembly of dipeptide and hydrophobic drug in hyaluronic acid through Schiff base reaction for the treatment of osteoarthritis
Source: Pharm Sci Adv. 2026 Jul 4;4:100133. doi: 10.1016/j.pscia.2026.100133 (PMC13356663; doi:10.1016/j.pscia.2026.100133)
Supplement: Multimedia component 1 [file mmc1.docx]

**Co-assembly of Dipeptide and Hydrophobic Drug in Hyaluronic Acid Through Schiff Base Reaction for the Treatment of Osteoarthritis**

Guixin Chen^a,^^†^, Qin Li^a,†^, Chen Yu^b,†^, Hao Liu^a^, Xueping Guo^a,c^, Aoli Wu^a,^*, Xiaoming Zhang^d,^*, Hengchang Zang^a,e,^*

^a^NMPA Center for Innovation and Research in Regulatory Science, School of Pharmaceutical Sciences, Cheeloo College of Medicine, Shandong University, Jinan 250012, Shandong, China

^b^Department of Pharmacy, Qilu Hospital of Shandong University, Jinan 250012, Shandong, China

^c^Bloomage GAG Biotechnology (Shenzhen) Corporation Limited, Shenzhen 518107, Guangdong, China

^d^School of Science, Optoelectronics Research Center, Minzu University of China, Beijing 100081, China

^e^State Key Laboratory of Discovery and Utilization of Functional Components in Traditional Chinese Medicine, Shandong University, Jinan 250012, Shandong, China

^†^These authors are considered as co-first authors.

*Corresponding authors:

wual@sdu.edu.cn (Aoli Wu); xmzhang@muc.edu.cn (Xiaoming Zhang); zanghcw@126.com (Hengchang Zang)

|  |
| --- |
| **Figure S1.** The roadmap for the Schiff base reaction of CDP with GA. |

| 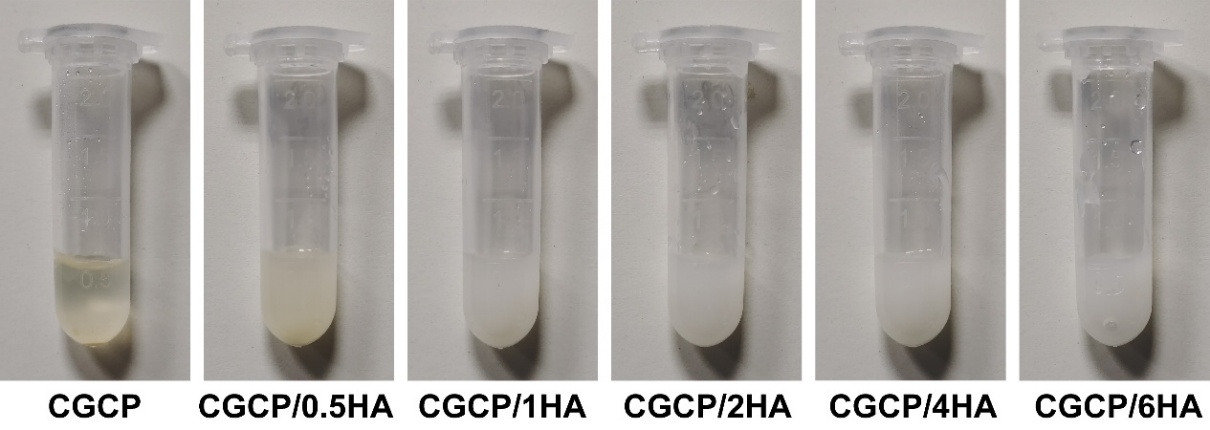 |
| --- |
| **Figure S2.** The images of CGCP and CGCP/HA at varying HA contents after one day of ageing. |

| 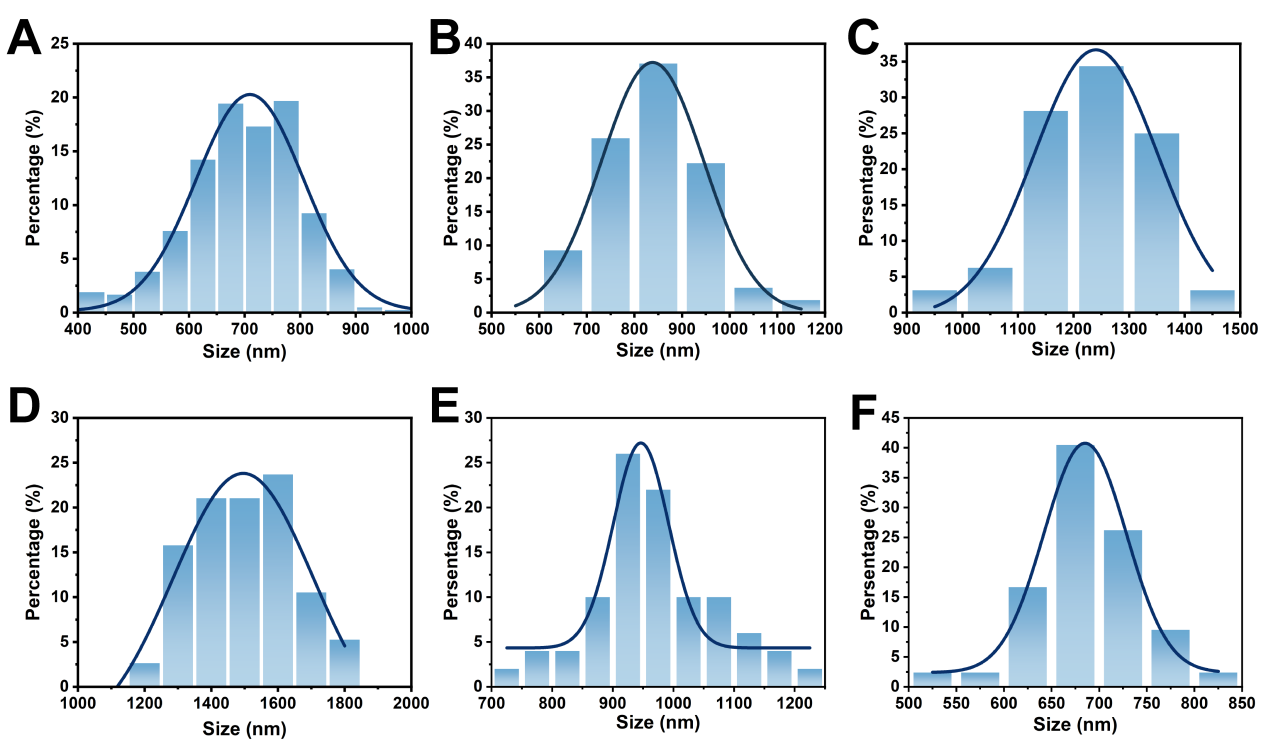 |
| --- |
| **Figure S3.** The histogram and fitted curves of particle size distributions for (A) CGCP, (B) CGCP/0.5HA, (C) CGCP/1HA, (D) CGCP/2HA, (E) CGCP/4HA, and (F) CGCP/6HA, based on statistical analysis of SEM measurements. |

| 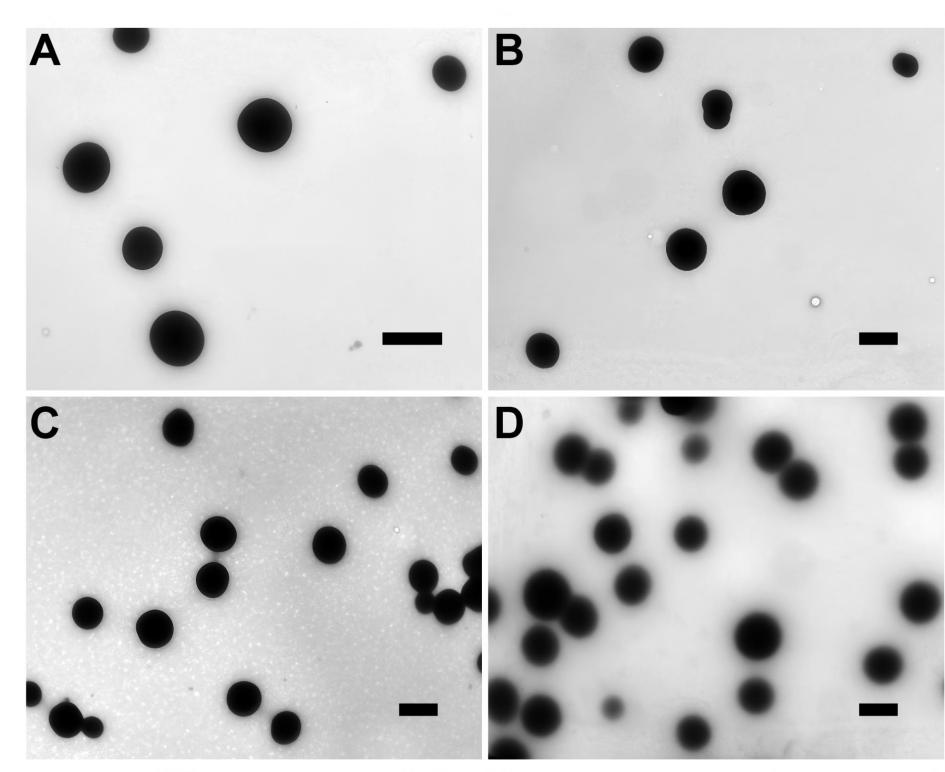 |
| --- |
| **Figure S4.** TEM images of (A) CGCP, (B) CGCP/0.5HA, (C) CGCP/1HA, and (D) CGCP/2HA, Scale bars, 1 μm. |

| 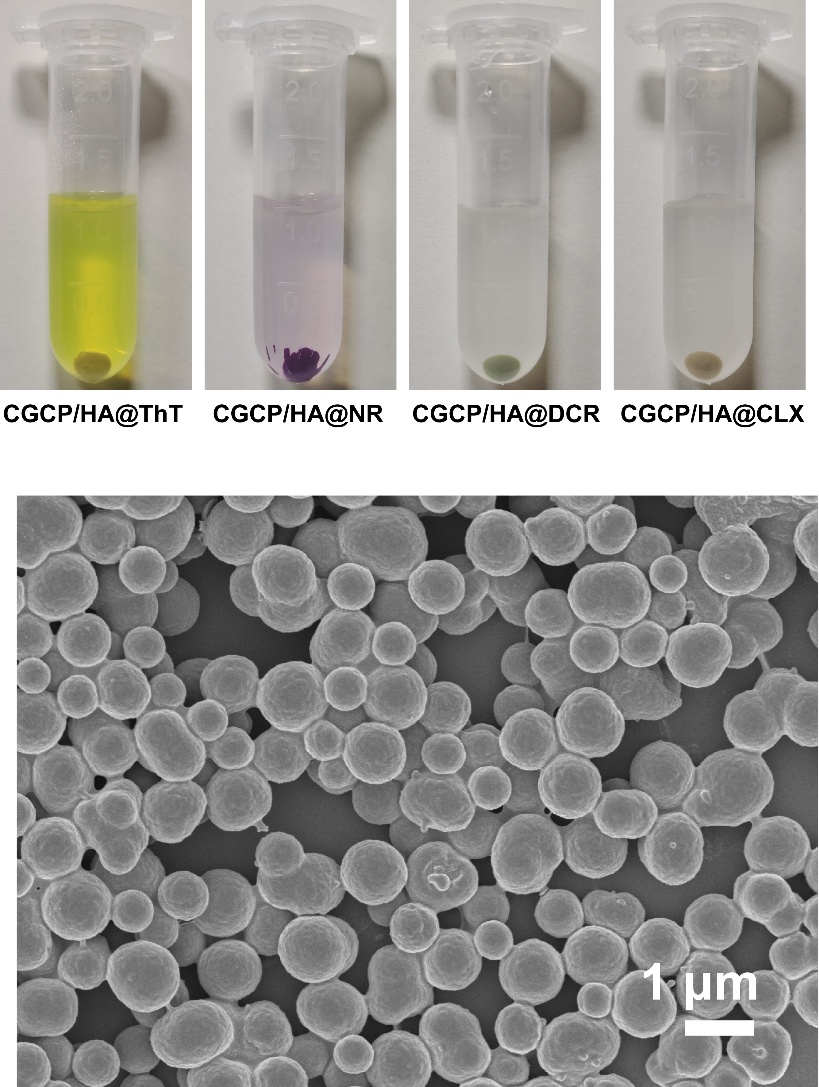 |
| --- |
| **Figure S5.** Photos of CGCP/HA@ThT, CGCP/HA@NR, CGCP/HA@DCR, and CGCP/HA@CLX. |

| 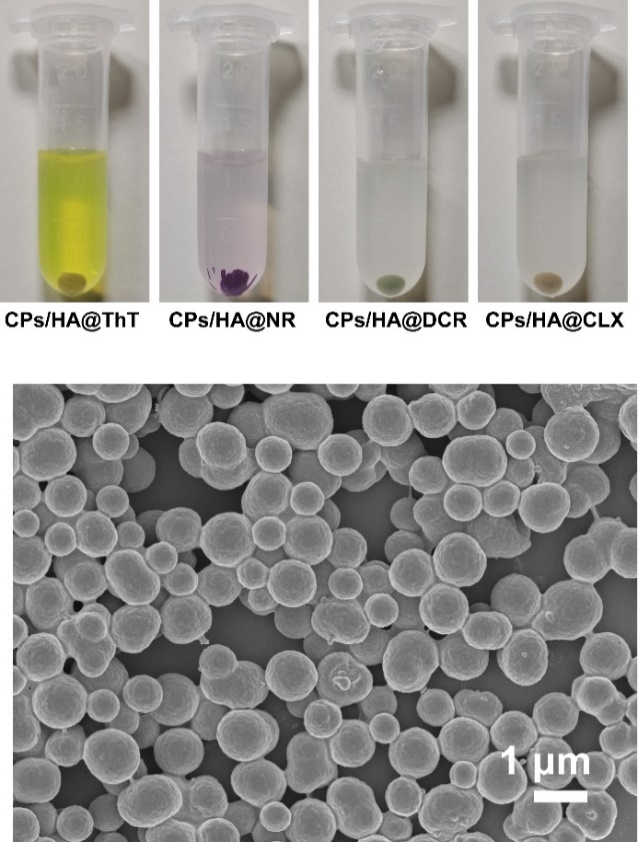 |
| --- |
| **Figure S6.** The SEM image of CGCP/HA@DCR+CLX. |


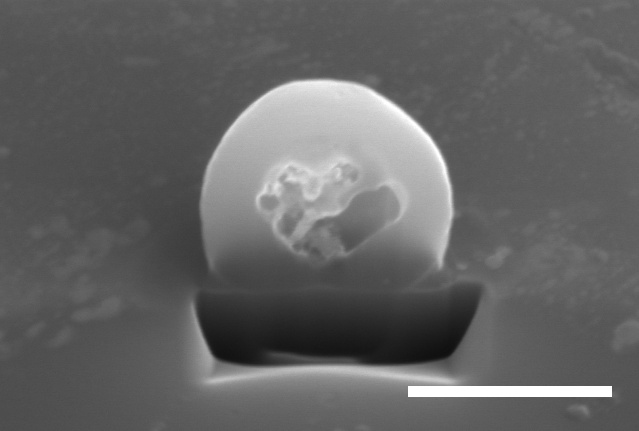


**Figure S7.** SEM image of CGCP/HA cut by ion beam.

| 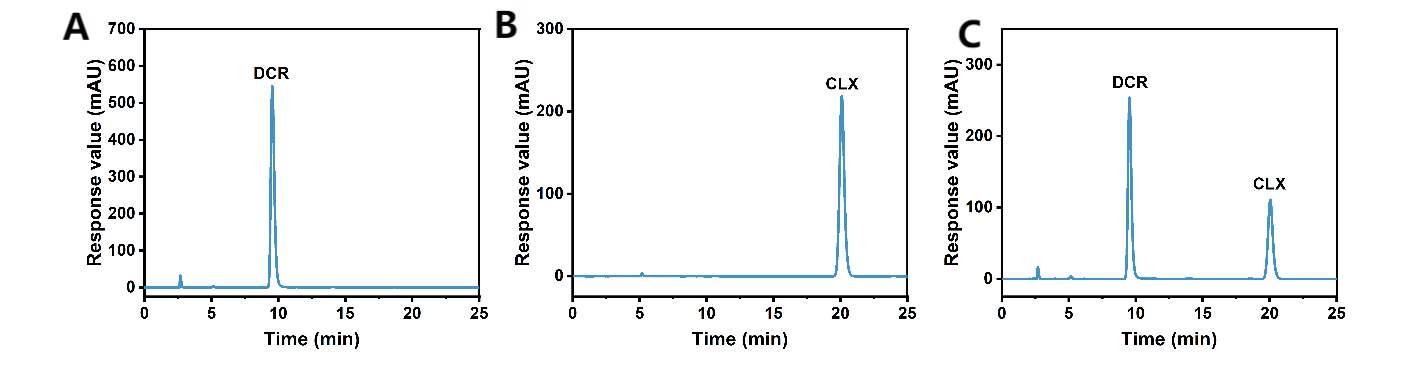 |
| --- |
| **Figure S8.** HPLC curves of DCR, CLX and mixed solution of DCR and CLX. |

| 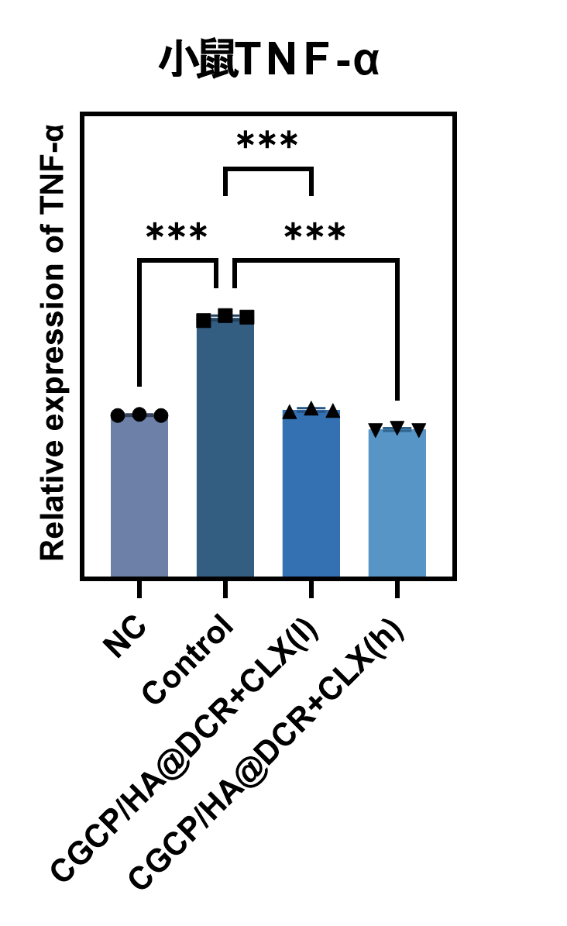 |
| --- |
| **Figure S9.** The levels of TNF-α in the cell supernatants following treatment with CGCP/HA@DCR+CLX were determined in RAW 264.7 cells (n=3). |


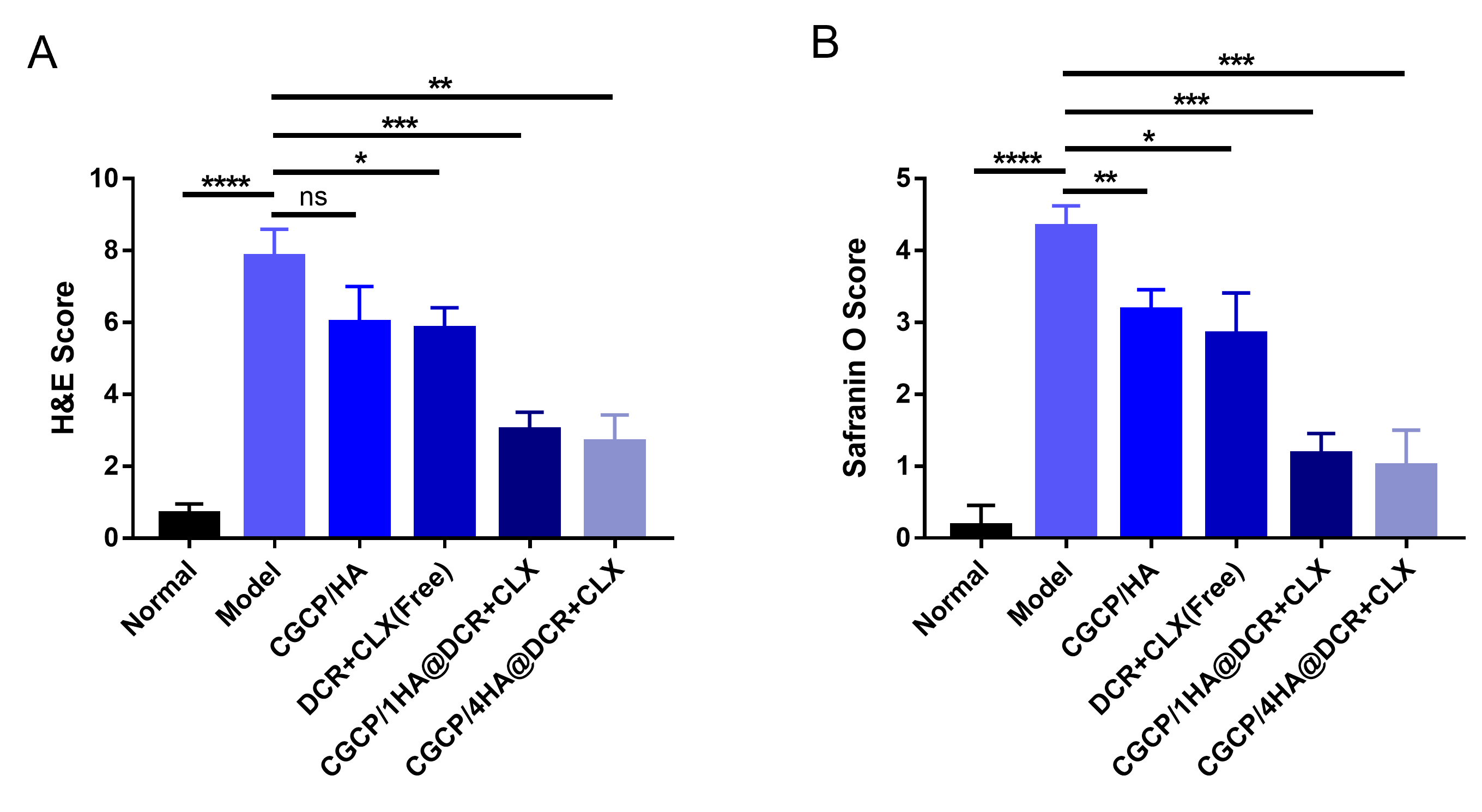


**Figure S10.** Histological staining scores. (A) H&E staining score. (B) Safranin O/Fast Green staining score. Data were expressed as mean ± SD, n = 3. ns, not significant; *P < 0.05, **P < 0.01, ***P < 0.001, ****P < 0.0001.
